# Molecular Analysis of Pathogenicity, Adhesive Matrix Molecules (MSCRAMMs) and Biofilm Genes of Coagulase-Negative Staphylococci Isolated from Ready-to-Eat Food

**DOI:** 10.3390/ijerph20021375

**Published:** 2023-01-12

**Authors:** Wioleta Chajęcka-Wierzchowska, Joanna Gajewska, Arkadiusz Józef Zakrzewski, Cinzia Caggia, Anna Zadernowska

**Affiliations:** 1 Department of Industrial and Food Microbiology, Faculty of Food Science, University of Warmia and Mazury in Olsztyn, 10-693 Olsztyn, Poland; 2 Department of Agriculture, Food and Environment (Di3A), University of Catania, Via Santa Sofia 100, 95123 Catania, Italy

**Keywords:** biofilm, coagulase-negative staphylococci, ready-to-eat food, pathogenicity, hemolytic activity, adherence, MSCRAMM, insertion elements IS*256/*IS*257*

## Abstract

This paper provides a snapshot on the pathogenic traits within CoNS isolated from ready-to-eat (RTE) food. Eighty-five strains were subjected to biofilm and slime production, as well as biofilm-associated genes (*icaA, icaD, icaB, icaC, eno, bap, bhp, aap, fbe, embP* and *atlE*), the insertion sequence elements IS*256* and IS*257* and hemolytic genes. The results showed that the most prevalent determinants responsible for the primary adherence were *eno* (57.6%) and *aap* (56.5%) genes. The *ica*ADBC operon was detected in 45.9% of the tested strains and was correlated to slime production. Moreover, most strains carrying the *ica*ADBC operon simultaneously carried the IS*257* insertion sequence element. Among the genes encoding for surface proteins involved in the adhesion to abiotic surfaces process, *atl*E was the most commonly (31.8%) followed by *bap* (4.7%) and *bhp* (1.2%). The MSCRAMMs, including *fbe* and *embp* were detected in the 11.8% and 28.2% of strains, respectively. A high occurrence of genes involved in the hemolytic toxin production were detected, such as *hla_yiD* (50.6%), *hlb* (48.2%), *hld* (41.2%) and *hla_haem* (34.1%). The results of the present study revealed an unexpected occurrence of the genes involved in biofilm production and the high hemolytic activity among the CoNS strains, isolated from RTE food, highlighting that this group seems to be acquiring pathogenic traits similar to those of *S. aureus*, suggesting the need to be included in the routine microbiological analyses of food.

## 1. Introduction

Staphylococci are a heterogeneous group of microorganisms that inhabit many environments due to their tolerance to unfavorable conditions. They constitute the natural microbiota of animals and humans, inhabiting the digestive tract and respiratory system, and occur as physiological fauna and flora on the skin and mucous membranes. As a result, they can easily spread from food handlers, surfaces in contact with hands and surfaces that come into contact with food during processing and packaging [1]. Due to this fact and high tolerance to environmental factors, they are a frequent microflora in ready-to-eat food (RTE) [2,3].

The pathogenicity of CoNS has been less considered than CoPS (e.g., *S. aureus*), which have been regarded as commensal microorganisms for years. Nevertheless, the continuous discoveries and updates on the species and subspecies have revealed a heterogeneous group, from non-pathogenic to facultatively pathogenic species, with varying levels of potential virulence. For now, coagulase-negative staphylococci (CoNS), are increasingly mentioned as a causative factor behind infections in individuals with a compromised immunity and they are considered as opportunistic pathogens, as have been reported by clinicians and microbiologists [4,5,6]. This has encouraged scientists to conduct more in-depth studies of the bacteria’s pathogenicity [7]. In general, CoNS isolates are poorer in the virulence determinants responsible for aggression. Nevertheless, the factors involved in the colonization may successfully support the bacterial-host interaction, a phenomenon that may be at least partly based on the multifunctional nature of various staphylococcal virulence factors known to exhibit redundant and overlapping functions. Despite the intensified research into clinical staphylococci, very few reports have focused on CoNS isolated from food, especially from ready-to-eat (RTE) products. The identification of the virulence factors, such as enterotoxins, has been described in our previous studies [8]. We have demonstrated that CoNS isolated from RTE food have a large repertoire of genes encoding superantigens. We have also highlighted that strains belonging to *S. epidermidis* have similar regulatory systems as the pathogenic *S. aureus* [9]. Moreover, the pathogenicity of CoNS is also associated with adhesion factors, the production of biofilms and hemolysins.

Biofilm formation is part of a normal staphylococcus life cycle in the environment [9], thanks to which planktonic cells stick to abiotic (e.g., polyethylene, steel, rubber, glass) or biotic (live tissue or abiotic surfaces covered with proteins) surfaces and proliferate and accumulate in multilayer cell clusters, embedded in special three-dimensional structures as mushrooms or towers, separated by fluid-filled channels [10]. There is much evidence to indicate that by adopting this lifestyle, bacteria have an advantage over planktonic cells. Indeed, biofilms protect microorganisms against disinfectants, proteases secreted by the host’s defense cells and environmental stressors [11]. This protection can contribute to the survival of staphylococci in the food processing environment, increasing the risk of cross-contamination [12,13]. Therefore, in order to develop new biofilm contamination-related strategies, it is necessary to better understand the CoNS biofilm growth at the molecular level.

Biofilm development is a several-stage process, in which bacteria first adhere to the surface to be colonized (primary adherence), and subsequently they gather in a multi-layer cellular architecture (accumulation phase) [14]. Many specific agents, such as autolysin AtlE [15], fibrinogen-binding protein—Fbe [16], fibronectin-binding protein—Embp [17] and autolysin Aae [18], take part in this process. These microbial surface components recognize the adhesive matrix molecules (MSCRAMMs—microbial surface components recognizing adhesive matrix molecules), which have been shown to act as extracellular matrix components at the early stages of biofilm formation. Since the ability to adhere to extracellular matrix or plasma proteins is closely associated with the host cell invasion, the biofilm formation has been related to the staphylococcus pathogenicity [19,20]. Most of the bacteria have no contact with the surface after the accumulation phase, but they remain in the biofilm owing to the inter alia polysaccharide intercellular adhesin (PIA), a homoglycan composed of β-1,6-linked N-acetylglucosamine residues [21,22]. The PIA is induced by the co-expression of the intercellular adhesin locus *ica*ADBC [23]. Data indicate that CoNS can form a biofilm also independently of the PIA. A significant role in the biofilm formation, in this case, is played by the extracellular matrix proteins, i.e., Aap [24].

Bacterial proteases become essential after adhesion. Their action involves cleaving the host’s proteins and enabling the microorganisms’ transition from an adhesive to an invasive phenotype [25,26]. The host’s cells can be invaded by means of a range of enzymes and cytolysins, which include hemolysins, that are classified into four types: alpha (α), beta (β), gamma (γ) and delta (δ) [27]. Toxin α is encoded by *hla*, and it acts as a cytotoxin against a wide range of human cells. The pathogenicity of the toxin is associated with hemolytic, dermal and neurotoxic effects [28]. Beta-hemolysin is a sphingomyelinase encoded by the *hlb* gene, and it is referred to as a “hot and cold” hemolysin, as incubation at temperatures under 10 °C boosts its cytolytic activity [29]. Toxin δ, encoded by the *hld* gene, is an exotoxin with a lysis capability for multiple cell types, including erythrocyte degradation [30].

As long as it is not clear whether all MSCRAMMs play a significant role in the biofilm formation and it is difficult to identify the differences between the invasive and commensal strains, as the virulence factors can be present in both. Recently, the sequential elements IS*256* have been found to be useful in distinguishing between the isolates [31].

Therefore, this present study was designed to detect the biofilm-forming capability, to determine the individual virulence markers involved in the process (*ica*A, *ica*D, *ica*B, *ica*C, *eno*, *bap*, *bhp*, *aap*, *fbe*, *emb*P, *atl*E) and to explore the relationship between the biofilm-forming capability and the presence of IS*256/*IS*257* in the strains isolated from RTE food. Moreover, a correlation between the biofilm-forming capability and the hemolytic activity and the species was also performed.

## 2. Materials and Methods

### 2.1. Staphylococci Strains

Eighty-five strains of coagulase-negative staphylococci isolated from 198 ready-to-eat food samples, as previously described, were investigated [8]. Briefly, the strains were isolated from sushi, salads, fresh squeezed juices, hamburgers, beef tartar and salmon tartar obtained from 11 randomly selected bars and restaurants in Olsztyn, Poland. The isolation of the strains was performed using standard microbiological methods, the identification was performed using VITEK^®^ MS (bioMérieux, Marcy l’Étoile, France), as previously described [8,32], and confirmed by the *tuf* gene sequencing, according to Li et al., 2012 [33].

The PCR products were resolved by electrophoresis and purified using the Clean-Up purification kit (A&A Biotechnology, Gdynia, Poland). The concentration and purity were measured using a DeNovix DS-11 spectrophotometer (DeNovix Inc., Wilmington, DE, USA). The PCR products were sequenced through the Sanger method at Genomed S.A. (Warsaw, Poland), using the same primers as those used for the PCR (Table S1).

### 2.2. Detection of the Ability of the Slime Production by the Congo Red Agar (CRA) Method

The ability to produce slime was determined using the Congo Red Agar method, according to Mathur et al. (2006) [34], as previously described [35]. A black colony was considered as a slime producer whereas Bordeaux and red colonies were considered as non-producing strains. All the tests were performed in triplicate.

### 2.3. Biofilm Forming Ability Detection by the Microtiter Plate Method (MTP)

The ability to produce a biofilm was tested according to Stepanović et al. (2007) [36], as previously described [35,37]. The absorbance at the 570 nm wavelength was measured with a spectrophotometric microplate reader Varioscan LUX (Thermo Scientific, Waltham, MA, USA). The following criteria were used for the biofilm gradation in the staphylococcal strains: the non-biofilm producers (OD ≤ ODc); the weak biofilm producers (ODc < OD ≤ 2 × ODc); the moderate biofilm producers (2 × ODc < OD ≤ 4 × ODc); the strong biofilm producers (4 × ODc < OD). All the tests were performed in triplicate.

### 2.4. Detection of the Biofilm-Associated Genes

The following biofilm-associated genes: *ica*A, *ica*D, *ica*B*, ica*C, *eno, bap, bhp, aap, fbe, emb*P, *atl*E were amplified by PCR using specific primers (Table S1). The PCR products were visualized by electrophoresis on 1.5% agarose gels in a 1×TBE (Tris-borate-EDTA) buffer stained by 0.5 μg/mL of ethidium bromide (0.5 mg/mL; Sigma-Aldrich Corp., St. Louis, MO, USA) and visualized using the G-BOX F3 system (Syngene, Cambridge, UK). *S. aureus* ATCC 25923 (*ica*A and *ica*D genes), *S. epidermidis* ATCC 14990 (*atl*E, *fbe*) and *S. epidermidis* ATCC 35984 (*aap*, *emb*P, *bhp*, *ica*B, *ica*C) were used as the control strains.

### 2.5. Detection of the Hemolysin Genes

The identification of the hemolysin encoding genes was performed by Multiplex PCR (*hla_haem, hla/yidD* and *hlb*) and single PCR (*hld)* using specific primers (Table S1). The PCR conditions were as previously described by Nasaj et al. 2020. [38] and *S. heamolyticus* ATCC 29970 (*hla_haem*), *S. epidermidis* ATCC 12228 (*hla/yidD, hlb*) and *S. aureus* N315 (*hld*) were used as positive controls.

### 2.6. Detection of IS256/IS257

The IS*256/*IS*257* genes were investigated by the PCR amplification using specific primer sequences (Table S1). Each 25 μL of the PCR reaction mixture contained: 2 μL template DNA, 1 μL of each forward and reverse primers, 9 μL of sterile distilled water, and 12.5 μL of 2× Taq DreamTaq Green PCR Master Mix (2×) (ThermoFisher Scientific, Waltham, Massachusetts, USA) [39]. *S. epidermidis* ATCC 35983 and *S. epidermidis* ATCC 12228 were used as positive and controls, respectively.

### 2.7. Statistical Analysis

All statistical analyses were performed using GraphPad Prism software version 8.0 (GRAPH PAD software Inc, San Diego, CA, USA). To determine the relationship between the slime production, the biofilm formation and the presence of the tested genes, the chi-squared Pearson test was used. All correlation analyses were calculated using the Pearson correlation, the occurrence of the genes was marked as 1 when the gene was present and 0 when it was absent, in which case the results of the Pearson correlation are identical to the point-biserial correlation. The strains producing strong, moderate and weak biofilms were summed up and defined as “biofilm producers”. The results were considered statistically significant when *p* < 0.05. The correlation of the binary values (0/1) of the biofilm/slime and genetic determinants was calculated using the “cor” function from the software “R” (R version 3.6.1; https://www.r-project.org/ (accessed on 4 September 2022)); the significance was determined using the function “cor.test”. The significant correlations were visualized using the “corrplot” function from the “corrplot” package in the software “R”.

## 3. Results

### 3.1. Qualitative and Quantitative Analyses of the Biofilm Formation by the CoNS Isolates

The result of the biofilm formation of the CoNS isolates by the MTP (quantitative) and CRA (qualitative) methods are shown in Table 1. The biofilm formation, tested by MTP, showed that 53 of the 85 CoNS isolates (62.4%) were biofilm producers, including 44 isolates (51.8%) that were strong biofilm producers and five isolates (5.9%) that were moderate biofilm producers. In contrast, four (4.7%) and 32 (37.6%) CoNS isolates were considered weak and negative for the biofilm formation ability. Using the CRA method, a total of 30 isolates (35.3%) were classified as slime producers, and 55 isolates (64.7%) were categorized as negative for slime formation. The correlation between the slime production in the CRA method and the biofilm formation by MTP was not significant (*p* = 0.279).

All of the *S. xylosus*, *S. lentus* and *S. piscifermentas* strains showed the ability to form a strong biofilm, while *S. lugdenensis* exhibited the ability to form a weak biofilm. The species that showed the lowest ability for the biofilm production were *S. carnosus*, *S. saprophyticus* and *S. pasteuri* species (Table 1). The results of the Pearson correlation indicated a non-significant small positive relationship between the biofilm formation (MTP) and the species. Nevertheless, no significant correlation between the species and the slime-producing capacity (CRA) was observed (*p* < 0.05).

**Figure 1:**
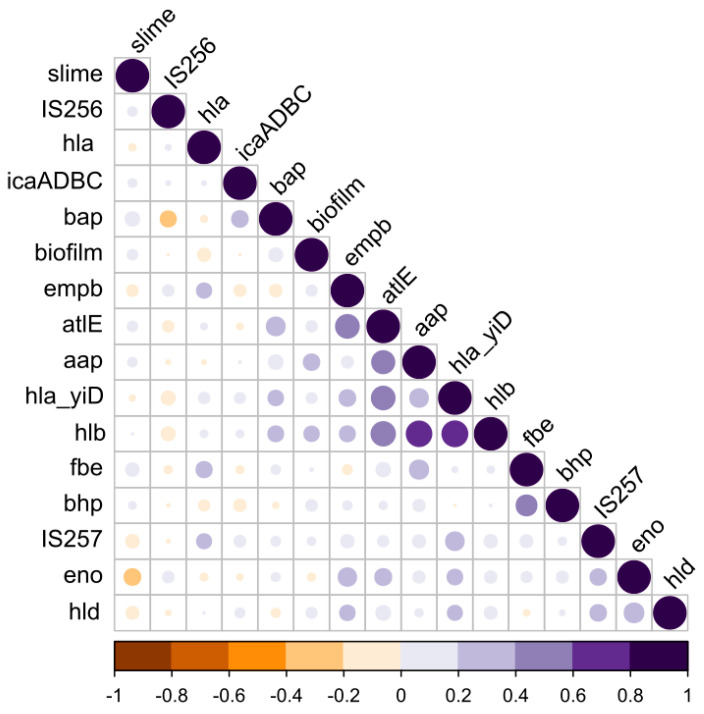
Correlation matrix indicating all correlations between the pairs of genetic or phenotypic determinants (biofilm and slime phenotypes). Shades of purple represent the positive correlations, while shades of orange represent the negative correlations.

Comparing the results obtained by the phenotypic biofilm formation with the *ica* operon’s detection, within the 39 *ica* positive CoNS strains, 22 (56.4%) were found to be biofilm producers, following the MTP method and 12 (30.8%) showed, following the CRA method, the ability to produce slime (Table 2). These results indicate the non-significant (*p* = 0.05), very small positive relationship between the ability to produce a biofilm detected by the MTP method and/or slime production and the presence of the *ica*ADBC operon (Figure 1).

### 3.2. Genetic Background Associated with the Biofilm and the Adherence in the CoNS Strains

The results on the presence of the genetic background responsible for the biofilm formation and adhesion among the CoNS strains are presented in Table 3. Among the genes responsible for the primary adherence, the most prevalent were *eno* (n = 49; 57.6%) and *aap* (n = 48; 56.5%). The genes encoding the fibrinogen binding protein Fbe and the fibronectin binding protein Embp were detected in 11.8% and 28.2% of the strains, respectively.

The presence of the *ica*ADBC operon was detected in 39 (45.9%) strains (Table 3), belonging to *S. simulans* (77.8%), *S. haemolyticus* (75%) *S. saprophyticus* (66.7%), *S. xylosus* and *S. piscifermentas* (50%), *S. warneri* (42.9%), *S. pasteuri* (40%), *S. epidermidis* (38.1%), *S. carnosus* (33.3%) and *S. petrasii* subsp. *petrasii* (25%). The results showed that the *ica*D and *ica*A genes had the highest prevalence in the CoNS isolates (29.4% and 25.9%, respectively), whilst *ica*B and *ica*C were found only in one isolate classified as *S. warneri* (Table S2).

Among the genes encoding the surface proteins influencing the adhesion to abiotic surfaces, the *atl*E (n = 27; 31.8%) was the mostly found. Overall, the prevalence of the *atl*E gene was found in all *S. lentus* and *S. lungudensis* strains, in 75% of the *S. haemolyticus* strains, in 66.7% of the *S. simulans*, in 57.1% of the *S. epidermidis*, in 40% of the *S. pasteuri* and in 7.1% of the *S. warneri* strains. The *bap* gene was found only in four *S. simulans* strains (44.4%) and in one *S. saprophyticus* strain (16.7%), while the *bhp* gene was detected only in one (16.7%) *S. saprophyticus* strain. The statistical analysis showed a relationship between the presence of *ica*ADBC (<0.0001), *eno* (<0.0001), *emb*P (*p* = 0.0006) *atl*E (*p* = 0.0124) genes and the CoNS species, whereas for the remaining genes, no correlation was found with the species.

### 3.3. Prevalence of the Hemolysin Genes and the Insertion Elements IS256/257 among the CoNS Strains

The distribution of the hemolysin genes among the CoNS strains is reported in Table 4. The results showed a high frequency of the hemolytic activity related genes among the CoNS strains from RTE food. In detail, 43 CoNS strains (50.6%) showed *hla_yiD*, 41 strains (48.2%) showed *hlb*, 35 strains (41.2%) showed *hld* and 29 strains (34.1%) showed the presence of the *hla_haem* gene (Table 4). The presence of the *hlb* (*p* = 0.0207) and *hla_yiD* (*p* = 0.0025) genes was strongly related to the CoNS species, which was statistically significant (*p* ≤ 0.05).

All hemolysin genes were detected only in six strains (7.1%) belonging to the species: *S. epidermidis*, *S. haemolyticus*, *S. petrasii* subsp. *petrasii*, *S. simulans and S. warneri* (n = 2). One type of hemolysin was found in 22 strains (25.9%), two types in 19 (22.4%) strains, and 22 (25.9%) strains were found positive for three hemolysin type genes (Table 5). The most common combination was *hla_yiD+hlb+hld*, observed in 13 (15.3%) strains belonging to *S. epidermidis* (n = 11) and *S. simulans* (n = 2) species (Table S3).

The distribution of the insertion sequence elements IS*256/IS257* and the hemolysin encoding genes (*hla*, *hla_yiD*, *hlb*, *hld*) among the *ica*^+^ and *ica^−^* isolates are shown in Table 5. The statistical analyses indicated no significant correlation between these genetic elements in the CoNS tested strains.

Moreover, the correlation matrix indicated that all of the hemolysin genes were correlated with *emb*P. Finally, a positive correlation within the *hld, hla_yiD, hlb* and *eno* genes was revealed.

### 3.4. Phenotype vs. the Genotype Correlations

The question of the phenotype–genotype and the gene–gene correlations is a complex issue, given the relatively large number of resistance and virulence genes that could be compared. To systematically detect all such associations in an unbiased way, we analyzed all potential pairs of variables in the contingency tables, and statistically assessed their dependence by Fisher’s exact test. In addition, using a pseudo-numeric binary matrix (in which the presence of the genes and biofilm/slime production ability is marked as 1 and the absence as 0) we estimated the correlation coefficients among all potential pairs (Figure 1). These analyses highlighted that the ability to form a biofilm by the CoNS is correlated to the genetic determinants, as *aap*, *atl*E, *hla_yiD*, *hlb*, *bhp* and *bap*, while it is not correlated to *ic*aADBC. However, the slime production capacity is correlated to *ica*ADBC. Interestingly, we also observed that most strains carrying *ica*ADBC, simultaneously, carry IS*257* in their genomes.

## 4. Discussion

The ability to form a biofilm is one of the most important virulence factors in staphylococci [40]. In the present study, the majority (62.4%) of the CoNS strains isolated from RTE food were capable of forming a biofilm, and 41.7% were classified as strong or moderate biofilm producers. Furthermore, different biofilm-forming capacities were observed among the strains, even belonging to the same species. The highest percentage of strains with a strong and moderate biofilm-forming capability was observed in the species: *S. xylosus* (100%), *S. lentus* (100%), *S. piscifermentas* (100%), *S. simulans* (77.8%) and *S. warneri* (57.1%).

It is relevant to highlight that among the CoNS, strains belonging to *S. xylosus*, *S. carnosus* and *S. warneri,* they are commonly exploited, as starter cultures, in ripened food production, e.g., cheese and fermented meat [41,42]. For this reason, due to its positive link with food fermentation processes, the biofilm production can be perceived as a desired feature, as both the adhesion and the biofilm formation can increase the starter assertiveness towards autochthonous microbiota, inducing, concomitantly, a resistance to the colonization in a specific ecological niche. Moreover, the biofilms provide physical protection to bacteria against stressors, including anti-microbial substances. However, given the increasing role of the CoNS in inducing human infections, most recently they tend to be perceived as potential pathogens. Therefore, it is extremely important to provide data characterizing the strains and demonstrate a link among the various virulence factors. Such a link among the virulence factors, as biofilm formation and hemolytic activity, as demonstrated in the present study, plays a crucial role in identifying the pathogenic strains.

Staphylococci, which are *ica*-positive, i.e., can synthesize the PIA, often exhibit the ability to produce slime on agar with Congo red [43]. The current study did not show any statistically significant correlation between the ability to produce slime and the presence of the *ica* operon genes. This can stem from numerous factors, one of them being the absence of a regulatory gene necessary for the expression of a phenotypic trait or from a mutation. The findings of this and our earlier research emphasize the importance to deeply explore the biofilm formation mechanism, independently from the *ica* presence in staphylococci [35].

Therefore, the role of MSCRAMMs in biofilm formation within the CoNS was also evaluated. Various MSCRAMMs were found in the CoNS isolated from RTE food, such as the biofilm-associated protein (*bap*), laminin-binding protein (*eno*), that exhibit a strain-specific variability, as previously reported by other authors [44,45,46]. Bacteria surface proteins, such as cell wall-anchored proteins (CWA), are regarded as virulence factors among gram-positive bacteria pathogens, and they play a key role in the microbial adherence to the host’s tissues, avoiding the host’s defense systems and biofilm formation [47]. The current study confirmed that a large majority of *S. epidermidis* isolated from RTE food contained the *atl*E, *aap* and *emb*P genes, as previously described for *S. epidermidis* isolated from blood infections associated with catheter and prosthetic joint infections (PJIs), or for the commensal *S. epidermidis*, for which the proteins are important, both in infection and in colonization [48]. Moreover, these determinants were found, not only in *S. epidermidis*, regarded as a potential human pathogen, but also in strains of *S. lentus*, *S. haemolyticus* and *S. lugdenensis*. The latest discoveries and updates concerning the CoNS species and subspecies confirmed this group as non-uniform, from non-pathogenic to potentially pathogenic, with different virulence levels [49]. Recent reports have highlighted that some species, such as *S. lugdunensis,* due to their higher virulent potential, are regarded as highly virulent pathogenic bacteria [50]. *S. lugdunensis* can cause very acute and destructive endocarditis (IE), with a higher mortality rate than other CoNS species, which generally cause less severe infections [51].

Compared to *S. aureus*, the CoNS have been studied less extensively. However, these species deserve special attention because of their growing importance, both from the clinical perspective and as a food-affecting agent, due to the multiple virulence factors. The surface colonization and the biofilm formation in the CoNS has been regarded as the main virulence factors because the heterogeneity of these bacteria in the biofilms is known to contribute to their survival, with emphasis on the viable but nonculturable (VBNC) cells and small colony variants (SCVs). Furthermore, the antibiotic resistance and enterotoxin production, which we have described in our earlier papers, also contribute to their virulence [2,3,8,52]. Given the toxin-formation potential and the observed increasing antibiotic and disinfectant resistance among the CoNS, the ability to form a strong biofilm observed in this study is not a cause for optimism. The cells in a biofilm exhibit a much greater tolerance to anti-microbial agents [53] and can easily survive adverse conditions during food processing and storage. Due to the high hemolytic activity observed in the CoNS isolated from RTE food, we can conclude that the CoNS should not be omitted in food microbiological analyses.

The current study also analyzed the link between the biofilm-forming capability and the hemolytic activity, finding a correlation between the biofilm-formation capability and the presence of the *hla_yiD* and *hlb_epi* genes. The hemolysin-encoding genes were also correlated to many genetic determinants responsible for the adhesion, such as the fibronectin-binding protein (*emb*P), laminin-binding protein (*eno*), biofilm external matrix protein (*aap*) and adhesion to abiotic surfaces (*atl*E). Moreover, the presence of the *hla_yiD* and *hlb* genes was correlated to IS*257*, as *hla* to IS*256.* A significant correlation between the presence of IS*256* and the hemolysin production has been recently observed by Nasaj et al. (2020) [38], within clinical isolates of the CoNS. Furthermore, a correlation between the biofilm formation and the hemolysin production in clinical strains was also demonstrated by Koskela et al. (2009) [54]. In addition, similar findings were observed by Huseby et al., who showed how beta-toxin stimulates the biofilm formation [55] within *S. aureus,* consistently with the current observations on the CoNS strains. Hemolysins, which belong to a group of cytolysins, are among the factors affecting the pathogenic potential of staphylococci [56].

The CoNS isolated from RTE food exhibited enough virulence and strategy factors to act as opportunistic pathogens, determined by the host’s specific conditions and by specific species- and strain-dependent traits. The CoNS clinical isolates contribute to the overall morbidity, mortality and socio-economic costs, being a non-solved medical issue likely to become exacerbated in the future. Finally, the alleged role of the CoNS as a dormant reservoir of antibiotic resistance, enterotoxicity and virulence factors, is still an underestimated issue, requiring greater scientific attention. All of this should induce food technologists to regard the resistant and virulent CoNS isolates as food pathogens, which should remain under supervision and control. Many CoNS pathogenicity determinants raise concerns, especially since their genes can be transmitted by a horizontal transfer to the *S.aureus* strains, thereby increasing their pathogenicity.

## 5. Conclusions

This study is the first study to be conducted on the CoNS isolated from RTE food to show a correlation between the biofilm formation and hemolytic activity. It has been demonstrated that hemolysins are correlated to multiple genes responsible for biofilm formation, apart from the PIA, rather than with *ica*ADBC. Furthermore, the insertion sequences, such as IS*256* and IS*257*, are associated with the slime production and some hemolysins, whereas the presence of those elements is not correlated with the biofilm-forming capability. The ability of the CoNS isolated from RTE food to form a strong biofilm, as observed in this study, as well as a potentially high hemolytic activity, indicates that the CoNS acquires characteristic features increasingly typical of the pathogenic *S. aureus*. More in-depth focus on these microorganisms is needed, especially since the coagulase-negative staphylococci are still not contemplated into routine microbiological food analyses.

## Figures and Tables

**Table 1:** Biofilm formation of the tested CoNS by the quantitative (MTP) and qualitative (CRA) methods.

Species	Biofilm Formation (MTP)				Slime Production (CRA)	
	Strong	Moderate	Weak	No Biofilm	Positive	Negative
*S. epidermidis* (n = 21)	10 (47.6%)	1 (4.8%)	0	10 (47.6%)	6 (28.6%)	15 (71.4%)
*S. warneri* (n = 14)	8 (57.1%)	1 (7.1%)	2 (14.3%)	3 (21.4%)	2 (14.3%)	12 (85.7%)
*S. carnosus* (n = 9)	2 (22.2%)	0	0	7 (77.8%)	3 (33.3%)	6 (66.7%)
*S. simulans* (n = 9)	7 (77.8%)	0	0	2 (22.2%)	7 (77.8%)	2 (22.2%)
*S. xylosus* (n = 8)	8 (100%)	0	0	0	6 (75%)	2 (25.0%)
*S. saprophyticus* (n = 6)	1 (16.7%)	1 (16.7%)	0	4 (66.7%)	1 (16.7%)	5 (83.3%)
*S. pasteuri* (n = 5)	2 (40.0%)	0	0	3 (60.0%)	0	5 (100%)
*S. heamolyticus* (n = 4)	0	1 (25.0%)	1 (25.0%)	2 (50.0%)	1 (25%)	3 (75.0%)
*S. petrasii* subsp. *petrasii* (n = 4)	2 (50.0%)	1	0	1 (25.0%)	2 (50%)	2 (50.0%)
*S. lentus* (n = 2)	2 (100%)	0	0	0	1 (50%)	1 (50.0%)
*S. piscifermentas* (n = 2)	2 (100%)	0	0	0	0	2 (100%)
*S. lugdenensis* (n = 1)	0	0	1 (100%)	0	1 (100%)	0
Total (n = 85)	44 (51.8%)	5 (5.9%)	4 (4.7%)	32 (37.6%)	30 (35.3%)	55 (64.7%)

**Table 2:** Comparison of the *ica* operon detection and the biofilm and slime production within the CoNS strains.

Biofilm Formation (MTP)		*ica*^+^ (n = 39)		*ica*^−^ (n = 46)		Total (n = 85)	
		n	(%)	n	(%)	n	(%)
strong		20	(51.3)	24	(52.1)	44	(51.8)
moderate		1	(2.6)	4	(8.7)	5	(5.9)
weak		1	(2.6)	3	(6.5)	4	(4.7)
no biofilm		13	(33.3)	19	(4.1)	32	(37.6)
**Slime production CRA**		**n**	**(%)**	**n**	**(%)**	**n**	**(%)**
positive		12	(25.6)	18	(41.3)	30	(35.3)
negative		23	(56.4)	32	(69.6)	55	(64.7)

n-number of strains.

**Table 3:** Detection of the biofilm-associated genes among the CoNS staphylococci.

Species	No. of Strains	*ica*ADBC	*bap*	*eno*	*aap*	*fbe*	*bhp*	*embP*	*atl*E	Biofilm Positive	Slime Production
*S. epidermidis*	21	8 (38.1%)	0	20 (95.2%)	15 (71.4%)	2 (9.5%)	0	12 (57.1%)	12 (57.1%)	11 (52.4%)	6 (28.6%)
*S. warneri*	14	6 (42.9%)	0	3 (21.4%)	6 (42.9%)	1 (7.1%)	0	3 (21.4%)	1 (7.1%)	11 (78.6%)	2 (14.3%)
*S. carnosus*	9	3 (33.3%)	0	6 (66.7%)	2 (22.2%)	0	0	0	0	2 (22.2%)	3 (33.3%)
*S. simulans*	9	7 (77.8%)	4 (44.4%)	4 (44.4%)	6 (66.7%)	2 (22.2%)	0	0	6 (66.7%)	7 (77.8%)	7 (77.8%)
*S. xylosus*	8	4 (50%)	0	1 (12.5%)	6 (75%)	1 (12.5%)	0	0	0	8 (100%)	6 (75%)
*S. saprophyticus*	6	4 (66.7%)	0	6 (100%)	3 (50%)	2 (33.3%)	1 (16.7%)	0	0	2 (66.7%)	1 (16.7%)
*S. pasteuri*	5	2 (40%)	0	1 (20%)	2 (40%)	0	0	2 (40%)	2 (40%)	2 (40%)	0
*S. heamolyticus*	4	3 (75%)	0	3 (75%)	3 (75%)	1 (25%)	0	4 (100%)	3 (75%)	2 (50%)	1 (25%)
*S. petrasii* subsp. *petrasii*	4	1 (25%)	0	1 (25%)	0	0	0	1 (25%)	0	3 (75%)	2 (50%)
*S. lentus*	2	0	0	2 (100%)	2 (100%)	1 (50%)	0	2 (100%)	2 (100%)	2 (100%)	1 (50%)
*S. piscifermentas*	2	1 (50%)	0	2 (100%)	2 (100%)	0	0	0	0	2 (100%)	0
*S. lugdenensis*	1	0	0	0	1 (100%)	0	0	0	1 (100%)	1 (100%)	1 (100%)
Total	85	39 (45.9%)	4 (4.7%)	49 (57.6%)	48 (56.5%)	10 (11.8%)	1 (1.2%)	24 (28.2%)	27 (31.8%)	53 (62.4%)	30 (35.3%)

**Table 4:** Prevalence of the hemolysin encoding genes and the insertion sequences IS*256* and IS*257* among the CoNS.

Species	No. of Strains	*hla_haem*	*hla_yiD*	*hlb*	*hld*	IS*256*	IS*257*
*S. epidermidis*	21	5 (23.8%)	14 (66.7%)	14 (66.7%)	16 (76.2%)	14 (66.7%)	19 (90.5%)
*S. warneri*	14	8 (57.1%)	5 (35.7%)	6 (42.9%)	8 (57.1%)	6 (42.9%)	13 (92.9%)
*S. carnosus*	9	0	5 (55.6%)	3 (33.3%)	1 (11.1%)	6 (66.7%)	8 (88.9%)
*S. simulans*	9	3 (33.3%)	7 (77.8%)	6 (66.7%)	3 (33.3%)	1 (11.1%)	9 (100%)
*S. xylosus*	8	0	0	0	1 (12.5%)	7 (87.5%)	5 (62.5%)
*S. saprophyticus*	6	2 (33.3%)	0	0	3 (50.0%)	2 (33.3%)	5 (83.5%)
*S. pasteuri*	5	3 (60.0%)	4 (80.0%)	2 (40.0%)	1 (20.0%)	2 (40.0%)	5 (100%)
*S. heamolyticus*	4	4 (100%)	3 (75.0%)	3 (75.0%)	1 (25.0%)	3 (75.0%)	4 (100%)
*S. petrasii* subsp. *petrasii*	4	3 (75.0%)	1 (25.0%)	2 (50.0%)	1 (25.0%)	1 (25.0%)	2 (50.0%)
*S. lentus*	2	0	2 (100%)	2 (100%)	0	2 (100%)	2 (100%)
*S. piscifermentas*	2	1 (50.0%)	1 (50.0%)	2 (100%)	0	0	2 (100%)
*S. lugdenensis*	1	0	1 (100%)	1 (100%)	0	1 (100%)	0
Total	85	29 (34.1%)	43 (50.6%)	41 (48.2%)	35 (41.2%)	45 (52.9%)	74 (87.1%)

**Table 5:** Comparison of the prevalence rates of the genes involved in the hemolysin production, *ica*ADBC operon and insertion elements.

Hemolysin Genes	*ica* ^+^		*ica* ^−^		Total (n = 85)	
	No	(%)	No	(%)	No	(%)
*hla* ^+^	14	(48.3)	15	(51.7)	29	(34.1)
*hla_yiD* ^+^	22	(51.1)	21	(48.8)	43	(50.6)
*hlb* ^+^	19	(46.3)	22	(53.7)	41	(48.2)
*hld* ^+^	19	(54.3)	16	(45.7)	35	(41.2)
Insertion sequences	No	(%)	No	(%)	No	(%)
IS*256*^+^	21	(46.7)	24	(53.3)	45	(52.9)
IS*257*^+^	35	(47.3)	39	(52.7)	74	(87.1)

## Data Availability

Not applicable.

## Supplementary Materials

The following supporting information can be downloaded at: https://www.mdpi.com/article/10.3390/ijerph20021375/s1. Table S1. List of the primer sequences used in the PCR reactions, Refs. [57,58,59,60,61,62] are cited in Supplementary Materials. Table S2. The results of the *ica* genes’ presence in the CoNS. Table S3. Hemolysin profiles of the CoNS strains isolated from ready-to-eat food. Table S4. Detailed characteristic of all the strains used in the study.
