# Supplementary material for: Molecular Analysis of Pathogenicity, Adhesive Matrix Molecules (MSCRAMMs) and Biofilm Genes of Coagulase-Negative Staphylococci Isolated from Ready-to-Eat Food
Source: Int J Environ Res Public Health. 2023 Jan 12;20(2):1375. doi: 10.3390/ijerph20021375 (PMC9859056; doi:10.3390/ijerph20021375)
Supplement: Supplementary file 1 [file ijerph-20-01375-s001.zip › ijerph-2019079-supplementary.pdf]

Supplementary materials:

Table S1. List of primer sequences used in PCR reactions.

Gene	Primer Sequence	Annealing temp. (°C)	Amplicon size (bp)	References
icaA	F: TCTCTTGCAGGA GCAATCAA	55,5	188	[1]
	R: TCAGGCACTAACATCCAGCA			
icaD	F: ATGGTCAAGCCCAGACAGAG		198	
	R: CGTGTTTTCAACATTTAATGCAA			
icaB	F: CTGATCAAGAATTTAAATCACAAA	52	302	[2]
	R: AAAGTCCCATAAGCCTGTTT			
icaC	F: TAACTTTAGGCGCATATGTTTT		400	
	R: TTCCAGTTAGGCTGGTATTG			
bap	F: CCCTATATCGAAGGTGTAGAATTG	60	971	[3]
	R: GCTGTTGAAGTTAATACTGTACCTGC			
eno	F: ACGTGCAGCAGCTGACT	55	302	[4]
	R: CAACAGCATCTTCAGTACCTTC			
aap	F: AAACGGTGGTATCTTACGTGAA	60	466	[5]
	R: CAATGTTGCACCATCTAAATCAGCT			
bhp	F: CTACAAGTTCAGGTCAAGGACAAGG	60	1583	
	R: GCGTCGGCGTATATCCTTCAG			
fbe	F: CTACAAGTTCAGGTCAAGGACAAGG	60	495	[5]
	R: GCGTCGGCGTATATCCTTCAG			
embp	F: AGCGGTACAAATGTCAAT	57	455	[6]
	R: AGAAGTGCTCTAGCATCATCC			
atlE	F: CAACTGCTCAACCGAGAACA	55	682	
	R: TTTGTAGATGTTGTGCCCCA			
hla_haem	F: TGGGCCATAAACTTCAATCGC	60	72	[7]
	R: ACGCCACCTACATGCAGATTT			
hla_yiD	F: TTTCKCCACTTACACCMCC		160	
	R: GGAACAGGATCAAAGCCACCT			
hlb	F: TGGTGGCGTTGGTATTGTGA		541	
	R: ACCCCAAGATTTCACGGACC			
hld	F: ATGGCAGCAGATATCATTTC		444	
	R: CGTGAGCTTGGGAGAGAC			
IS256	F: AGTCCTTTTACGGTACAATG	54	762	[8]
	R: TGTGCGCATCAGAAATAACG			
IS257	F: CTATCTAAGATATGCATTGAG		577	
	R: TTAACTTGCTAGCATGATGC			
tuf	F: CCAATGCCACAAACTCGT	50	830	[9]
	R: CCTGAACCAACAGTACGT			

References:

1. Arciola, C.R.; Baldassarri, L.; Montanaro, L. Presence of icaA and icaD genes and slime production in a collection of Staphylococcal strains from catheter-associated infections. *J. Clin. Microbiol.* **2001**, *39*, 2151–2156.
2. Solati, S.M.; Tajbakhsh, E.; Khamesipour, F.; Gugnani, H.C. Prevalence of virulence genes of biofilm producing strains of Staphylococcus epidermidis isolated from clinical samples in Iran. *AMB Express* **2015**, *5*, 47.
3. Cucarella C., Solano C., Valle J., Amorena B., Lasa I., P.J.R. Bap, a Staphylococcus aureus Surface Protein Involved in Biofilm Formation Staphylococcus aureus Surface Protein Involved in Biofilm Formation. *Microbiology* **2001**, *183*, 2888–2896.
4. Tristan, A.; Ying, L.; Bes, M.; Etienne, J.; Vandenesch, F.; Lina, G. Use of multiplex PCR to identify Staphylococcus aureus adhesins involved in human hematogenous infections. *J. Clin. Microbiol.* **2003**, *41*, 4465–4467.
5. Rohde, H.; Burdelski, C.; Bartscht, K.; Hussain, M.; Buck, F.; Horstkotte, M.A.; Knobloch, J.K.M.; Heilmann,

C.; Herrmann, M.; Mack, D. Induction of Staphylococcus epidermidis biofilm formation via proteolytic processing of the accumulation-associated protein by staphylococcal and host proteases. *Mol. Microbiol.* **2005**, *55*, 1883–1895
6. Rohde, H.; Burandt, E.C.; Siemssen, N.; Frommelt, L.; Burdelski, C.; Wurster, S.; Scherpe, S.; Davies, A.P.; Harris, L.G.; Horstkotte, M.A.; et al. Polysaccharide intercellular adhesin or protein factors in biofilm accumulation of Staphylococcus epidermidis and Staphylococcus aureus isolated from prosthetic hip and knee joint infections. *Biomaterials* **2007**, *28*, 1711–1720.
7. Nasaj, M.; Saeidi, Z.; Asghari, B.; Roshanaei, G.; Arabestani, M.R. Identification of hemolysin encoding genes and their association with antimicrobial resistance pattern among clinical isolates of coagulase-negative Staphylococci. *BMC Res. Notes* **2020**, *13*, 4–9.
8. Chessa, D.; Ganau, G.; Spiga, L.; Bulla, A.; Mazzarello, V.; Campus, G.V.; Rubino, S. Staphylococcus aureus and Staphylococcus epidermidis Virulence Strains as Causative Agents of Persistent Infections in Breast Implants. **2016**.
9. Li, X.; Xing, J.; Li, B.; Wang, P.; Liu, J. Use of tuf as a target for sequence-based identification of Gram-positive cocci of the genus Enterococcus, Streptococcus, coagulase-negative Staphylococcus, and Lactococcus. *Ann. Clin. Microbiol. Antimicrob.* **2012**, *11*.

Table S2. The results of *ica* genes presence in CoNS.

Species	No. of	*ica*A	*ica*D	*ica*B	*ica*C
S. epidermidis	21	2 (9.5%)	6 (28.6%)	0	0
S. warneri	14	4 (28.6%)	1 (7.1%)	1 (7.1%)	1 (7.1%)
S. carnosus	9	2 (22.2%)	1 (11.1%)	0	0
S. simulans	9	7 (77.8%)	6 (66.7%	0	0
S. xylosus	8	1 (12.5%)	4 (50%)	0	0
S. saprophyticus	6	2 (33.3%)	2 (33.3%)	0	0
S. pasteuri	5	1 (20%)	1 (20%)	0	0
S. heamolyticus	4	1 (25%)	3 (75%)	0	0
S. petrasii subsp. petrasii	4	1 (25%)	0	0	0
S. lentus	2	0	0	0	0
S. piscifermentas	2	1 (50%)	1 (50%)	0	0
S. lugdenensis	1	0	0	0	0
**Total**	**85**	**22 (25.9%)**	**25 (29.4%)**	**1 (1.8%)**	**1(1.8%)**

Table S3. Hemolysins profiles in CoNS isolated from ready-to-eat food

Hemolysin genes	Strains no. (%)
hld	7 (8.2%)
hla_haem	6 (7.1%)
hlb	5 (5.9%)
hla_yiD	4 (4.7%)
hla_yiD+hlb	7 (8.2%)
hla_haem+hld	5 (5.9%)
hla_haem+hla_yiD	3 (3.5%)
hla_yiD+hld	2 (2.3%)
hla_haem+hlb	1 (1.2%)
hla_yiD+hlb+hld	13 (15.3%)
hla_haem+hla_yiD+hlb	8 (9.4%)
hla_haem+ hla_yiD+hld	1 (1.2%)
hla_haem+ hla_yiD+hld+hlb	6 (4.7%)

Table S4 Detailed characteristic of all the strains used in the study.

L.p	Identification MALDI TOF MS	biofilm	slime production	Biofilm associated genes	Hemolysin genes	Insertion sequences
1	*S. carnosus*	strong	no	*eno*	*hla_yiD*	IS256, IS257
2	*S. carnosus*	no biofilm	yes	-	*hlb*	IS256, IS257
3	*S. carnosus*	no biofilm	yes	*icaA*	-	IS256,
4	*S. carnosus*	no biofilm	no	*icaD, eno*	*hla_yiD*	IS256, IS257
5	*S. carnosus*	no biofilm	yes	*icaA*	*hla_yiD, hld*	IS257
6	*S. carnosus*	no biofilm	no	-	*hla_yiD*	IS257
7	*S. carnosus*	no biofilm	no	*eno*	*hla_yiD*	IS257
8	*S. carnosus*	no biofilm	no	*eno, aap*	*hlb*	IS256, IS257
9	*S. carnosus*	strong	no	*aap*	*hlb*	IS256, IS257
10	*S. epidermidis*	no biofilm	no	*icaA, eno*	*hla_yiD, hld*	IS256, IS257
11	*S. epidermidis*	strong	no	*eno, embP, atlE*	-	IS256
12	*S. epidermidis*	strong	no	*icaD, eno, aap*	*hla_yiD, hlb*	IS256, IS257
13	*S. epidermidis*	strong	no	*eno, aap*	*hla_yiD, hlb, hld*	IS256, IS257
14	*S. epidermidis*	strong	no	*icaD, eno, aap, embP,*	*hla_yiD, hlb, hld*	IS257
15	*S. epidermidis*	strong	no	*eno, aap, embP, atlE*	*hla_yiD, hlb, hld*	IS257
16	*S. epidermidis*	strong	no	*eno, aap, empB, atlE*	*hla_yiD, hlb, hld*	IS257
17	*S. epidermidis*	strong	no	*eno, embP, atlE*	*hla_yiD, hlb, hld*	IS257
18	*S. epidermidis*	no biofilm	no	*icaA, icaD, aap,*	*hlb*	-
19	*S. epidermidis*	no biofilm	yes	*icaD, eno, embP*	*hla_haem*	IS256, IS257
20	*S. epidermidis*	no biofilm	yes	*eno, aap, fbe, atlE*	*hla_haem*	IS256, IS257
21	*S. epidermmidis*	intermediate	yes	*aap*	*hla_yiD, hlb, hld*	IS256, IS257
22	*S. epidermmidis*	strong	no	*eno, aap, empB, atlE*	*hla_yiD, hlb, hld*	IS256, IS257
23	*S. epidermmidis*	strong	no	*aap, embP, atlE*	*hla_yiD, hlb, hld*	IS257
24	*S. epidermmidis*	no biofilm	no	*eno, aap, embP, atlE*	*hla_haem, hla_yiD,*	IS256, IS257
25	*S. epidermmidis*	no biofilm	no	-	*hld*	IS256, IS257
26	*S. epidermmidis*	no biofilm	no	*eno, embP*	*hla_haem, hld*	IS256, IS257
27	*S. epidermmidis*	no biofilm	no	*eno, aap, fbe, atlE*	*hla_haem, hld*	IS256, IS257
28	*S. epidermmidis*	strong	yes	*icaD, eno, aap*	*hla_yiD, hlb, hld*	IS256, IS257
29	*S. epidermmidis*	no biofilm	yes	*eno, aap, embP, atlE*	*hla_yiD, hlb, hld*	IS257
30	*S. epidermmidis*	no biofilm	yes	*icaD, eno, aap, embP,*	*hla_yiD, hlb, hld*	IS256, IS257
31	*S. haemolyticus*	no biofilm	no	*eno, aap, embP, atlE*	*hla_haem, hla_yiD,*	IS257
32	*S. haemolyticus*	weak	yes	*icaD, aap, fbe, embP,*	*hla_haem, hla_yiD,*	IS256, IS257
33	*S. haemolyticus*	intermediate	no	*icaD, eno, aap, embP,*	*hla_haem, hla_yiD,*	IS256, IS257
34	*S.heamolyticus*	no biofilm	no	*icaA, icaD, eno, embP*	*hla_haem*	IS256, IS257
35	*S. lentus*	strong	yes	*eno,aap,fbe,embP, atlE*	*hla_yiD, hlb*	IS256, IS257

36	*S. lentus*	strong	no	*eno, aap, embP, atlE*	*hla_yiD, hlb*	IS256, IS257
37	*S. lugdenensis*	weak	yes	*aap, atlE*	*hla_yiD, hlb*	IS256,
38	*S. pasteuri*	strong	no	*icaD, aap, embP, atlE*	*hla_yiD, hlb*	IS257
39	*S. pasteuri*	strong	no	*icaA, eno, embP*	*hla_haem, hla_yiD,*	IS256, IS257
40	*S. pasteuri*	no biofilm	no	-	*hla_haem, hla_yiD*	IS256, IS257
41	*S. pasteuri*	no biofilm	no	*aap, atlE*	*hla_haem, hla_yiD,*	IS257
42	*S. pasteuri*	no biofilm	no	-	-	IS257
43	*S. petrasii subsp.*	strong	no	*embP*	*hla_haem*	IS257
44	*S. petrasii subsp.*	no biofilm	yes	-	-	-
45	*S. petrasii subsp.*	strong	no	*icaA, eno*	*hla_haem, hla_yiD,*	IS256, IS257
46	*S. petrasii subsp.*	intermediate	yes	-	*hla_haem, hlb*	-
47	*S. piscifermentans*	strong	no	*icaA, icaD,eno, aap*	*hlb*	IS257
48	*S. piscifermrntans*	strong	no	*eno, aap, fbe*	*hla_haem, hla_yiD,*	IS257
49	*S. saprophyticus*	no biofilm	no	*icaA, eno*	-	IS256, IS257
50	*S. saprophyticus*	no biofilm	no	*icaD, eno*	*hla_haem*	IS256, IS257
51	*S. saprophyticus*	no biofilm	no	*icaD, eno*	*hld*	-
52	*S. saprophyticus*	no biofilm	no	*icaA, icaD, eno, aap,*	*hla_haem, hld*	IS257
53	*S. saprophyticus*	strong	yes	*eno, aap*		IS257
54	*S. saprophyticus*	intermediate	no	*eno, aap, fbe, bhp*	*hld*	IS257
55	*S. simulans*	strong	no	-	-	IS257
56	*S. simulans*	no biofilm	yes	-	-	IS256, IS257
57	*S. simulans*	strong	yes	*icaA, icaD, bap, eno,*	*hla_haem, hla_yiD,*	IS257
58	*S. simulans*	no biofilm	yes	*icaA, icaD, aap, atlE*	*hla_haem, hla_yiD,*	IS257
59	*S. simulans*	strong	yes	*icaA, icaD, eno, aap,*	*hla_yiD, hlb, hld*	IS257
60	*S. simulans*	strong	no	*icaA, icaD, bap, eno,*	*hla_yiD, hlb, hld*	IS257
61	*S. simulans*	strong	yes	*icaA, bap, eno, aap,*	*hla_yiD, hlb*	IS257
62	*S. simulans*	strong	yes	*icaA, icaD, bap, aap,*	*hla_yiD, hlb*	IS257
63	*S. simulans*	strong	yes	*icaA, icaD*	*hla_haem, hla_yiD*	IS257
64	*S. warneri*	strong	no	*icaA, eno, aap, embP,*	*hla_haem, hla_yiD,*	IS256, IS257
65	*S. warneri*	strong	yes	*icaA*	*hla_haem, hld*	IS256, IS257
66	*S. warneri*	strong	no	*icaA*	-	IS256, IS257
67	*S. warneri*	strong	no	*aap*	*hlb, hld*	IS256, IS257
68	*S. warneri*	strong	yes	*eno, embP*	*hla_haem, hld*	IS256, IS257
69	*S. warneri*	strong	no	*icaD, eno*	*hld*	IS257
70	*S. warneri*	strong	no	*icaC, icaB*	*hld*	IS257
71	*S. warneri*	weak	no	-	*hld*	IS257
72	*S. warneri*	weak	no	-	*hla_haem, hla_yiD,*	IS257
73	*S. warneri*	no biofilm	no	*icaA, aap, fbe*	*hla_haem, hla_yiD,*	IS257
74	*S. warneri*	no biofilm	no	*aap*	*hla_haem, hla_yiD,*	IS257

75	*S. warneri*	intermediate	no	*aap*	*hla_haem, hla_yiD,*	IS257
76	*S. warneri*	strong	no	*aap, embP*	*hla_haem*	IS256, IS257
77	*S. warneri*	no biofilm	no	*eno*	-	-
78	*S. xylosus*	strong	no	*icaA, icaD, eno, aap*	*hld*	IS256, IS257
79	*S. xylosus*	strong	no	*aap*	-	IS256, IS257
80	*S. xylosus*	strong	yes	*aap, fbe*	-	IS256, IS257
81	*S. xylosus*	strong	yes	*aap*	-	IS256
82	*S. xylosus*	strong	yes	*aap*	-	IS256
83	*S. xylosus*	strong	yes	*icaD*	-	IS256
84	*S. xylosus*	strong	yes	*icaD*	-	IS257
85	*S. xylosus*	strong	yes	*icaD, aap*	-	IS256, IS257
